# T-cell-specific mTOR deletion in mice ameliorated CD4^+^ T-cell survival in lethal sepsis induced by severe invasive candidiasis

**DOI:** 10.1080/21505594.2019.1685151

**Published:** 2019-10-31

**Authors:** Hao Wang, Guangxu Bai, Na Cui, Wen Han, Yun Long

**Affiliations:** a Department of Critical Care Medicine, Peking Union Medical College Hospital, Peking Union Medical College and Chinese Academy of Medical Science, Beijing, China; b Department of Clinical Laboratory, Peking Union Medical College Hospital, Peking Union Medical College, Chinese Academy of Medical Science; Beijing Key Laboratory for Mechanisms Research and Precision Diagnosis of Invasive Fungal Diseases, Beijing, China

**Keywords:** Lethal fungal sepsis, severe invasive candidiasis, CD4^+^ T cells, mammalian target of rapamycin, autophagy

## Abstract

The mammalian target of rapamycin (mTOR) pathway can mediate T-cell survival; however, the role of this pathway in T-cell survival during fungal sepsis is unclear. Here, we investigated the role of the mTOR pathway in CD4^+^ T-cell survival in a mouse model of rapidly progressive lethal sepsis induced by severe invasive candidiasis and explored the possible mechanism. The decrease in CD4^+^ T-cell survival following fungal sepsis was ameliorated in mice with a T-cell-specific mTOR deletion, whereas it was exacerbated in mice with a T-cell-specific tuberous sclerosis complex (TSC)1 deletion. To explore the mechanism further, we measured expression of autophagy proteins light chain 3B and p62/sequestosome 1 in CD4^+^ T cells. Both proteins were increased in T-cell-specific mTOR knockout mice but lower in T-cell-specific TSC1 knockout mice. Transmission electron microscopy revealed that T-cell-specific mTOR knockout mice had more autophagosomes than wild-type mice following fungal sepsis. CD4^+^ T-cell mTOR knockout decreased CD4^+^ T-cell apoptosis in fungal sepsis. Most notably, the T-cell-specific mTOR deletion mice had an increased survival rate after fungal sepsis. These results suggest that the mTOR pathway plays a vital role in CD4^+^ T-cell survival during fungal sepsis, partly through the autophagy–apoptosis pathway.

## Introduction

Candidemia is the most common invasive fungal disease worldwide [1]. Due mainly to the significant progress in transplantation procedures, the rapid development of cancer chemotherapy and immunotherapy and the broader use of corticosteroids, about 400,000 people globally develop candidemia every year, and the mortality approaches 40% [2–4]. To make matters worse, septic shock caused by *Candida albicans* is fatal, with a mortality rate approaching 90%, which is three times that of septic shock induced by bacteria [5]. The main reason is that most of the patients with candidemia are immunocompromised or in critical condition. Once septic shock occurs, it progresses rapidly, combined with severe multiple organ failure, and causes rapid death in more than half the patients within 7 days [6]. Accordingly, in recent years, some studies have attempted to improve the prognosis through immunomodulation combined with antifungal medication [7].

The host immune response to fungal infection occurs in a coordinated way via both the innate and adaptive immune pathways. The first line of defense is innate effector cells, mainly macrophages and neutrophils, and the second line of defense is the adaptive immune system, which involves mainly CD4^+^ T cells [2,8]. There is a well-documented state of T-cell survival that rapidly develops after bacterial sepsis, which is closely correlated to poorer outcomes of sepsis [9]; however, there are few studies on this phenomenon in fungal sepsis.

The mammalian target of rapamycin (mTOR) pathway is an evolutionarily conserved mechanism that primarily controls cell growth and metabolism [10,11]. It consists of two protein complexes, mTOR complex (mTORC)1 and mTORC2; mTORC1 is activated mainly through the phosphoinositide 3-kinase–AKT pathway. After its activation, mTORC1 phosphorylates S6 kinase (S6K) and the translational initiation factor 4E binding protein 1. mTORC1 function is negatively regulated by tuberous sclerosis complex (TSC)1 [12,13]. The mTOR signaling pathway is extensively involved in lymphocyte biology; numerous immune signals can activate the mTOR pathway, which in turn regulates lymphocyte development, activation and differentiation [14,15]. In addition, the mTOR signaling pathway plays an important role in the regulation of programmed cell death, namely autophagy and apoptosis [16].Recent studies have shown another critical role for the mTOR pathway in lymphocyte survival [17,18], but the underlying mechanisms are not clear. Our previous studies [19,20] found that the mTOR pathway influences the prognosis of Invasive Pulmonary Aspergillosis (IPA) through the regulation of CD8 + T cell differentiation. However, up to date the role of mTOR in invasive candidiasis is still unclear.

Autophagy is a protein-degradation system. Its main functions are to recycle proteins, remove damaged organelles, eliminate microorganisms, and act in antigen presentation [21]. Multiple studies have demonstrated that autophagy plays a protective role in several organs during sepsis, and recent work has shown that autophagy also plays a vital role in the survival of lymphocytes [22–24]. However, the relationship between lymphocyte survival and autophagy in fungal sepsis is not well documented. In the current study, we explored T-cell survival in mice with lethal *Candida* sepsis and investigated the possible underlying pathophysiological mechanisms.

## Materials and methods

### Mice

T-cell-specific *TSC1* and *mTOR* conditional knockout mice (*Lck-cre TSC1^loxp/loxp^* and *Lck-cre mTOR^loxp/loxp^*, respectively) were obtained by crossing *TSC1^loxp/loxp^* and *mTOR^loxp/loxp^* mice, respectively, with mice expressing *Cre* recombinase under the control of the T-cell-specific promoter Lck (lymphocyte-specific protein tyrosine kinase). *Lck-cre*-negative *mTOR^loxp/loxp^* littermates served as the control animals. Four-to-five-week-old male *Lck-cre mTOR^loxp/loxp^* (lck-mTOR), *Lck-cre TSC1^loxp/loxp^* (lck-TSC1), and *Lck-cre*-negative *mTOR^loxp/loxp^* (wild type) mice were used for *in vivo* experiments, and there were 6 mice in each group. The *TSC1^loxp/loxp^, mTOR^loxp/loxp^* and *Lck-cre* mice were kindly provided by Dr. Yong Zhao (State Key Laboratory of Biomembrane and Membrane Biotechnology, Institute of Zoology, Chinese Academy of Sciences, Beijing, China). All mice were acclimated to a 12-h day/night cycle under specific pathogen-free conditions with food for at least 1 week before the experiments.

### *Mouse infection and* C. *albicans treatment*

We constructed a mouse model of *C. albicans* bloodstream infection by administering an intravenous tail injection of 100 µl of 10^6^ colony-forming units (cfu) of *C. albicans* strain SC5314 [25]. The control groups were injected with an equivalent dose of saline. The mice were killed at 12 h after *C. albicans* injection, and their kidneys, spleens, livers and lungs were removed. The successful establishment of our mouse model of severe invasive *C. albicans* multiple organs infection was confirmed by Gomori’s methenamine silver (GMS) staining.

### Histopathology

Kidneys, spleens, livers and lungs were fixed in 10% neutral-buffered formalin and stored at 4°C until required. The tissues were embedded in paraffin, cut into 4-µm sections and stained with GMS. Stained slides were scanned using a Pannoramic slide scanner and evaluated for intralesional fungal burden.

### Lymphocyte isolation and cell counting

Spleens were surgically removed from anesthetized mice and gently pressed with slide glasses. They were then washed with PBS, and red blood cells were lysed with ammonium-chloride-potassium lysis buffer (BD). Centrifuged lymphocytes were resuspended in RPMI-1640 medium. The number and viability of the lymphocytes resuspended in RPMI-1640 medium were quantified with a TC20 automated cell counter (Bio Rad).

### Surface marker staining

The pellets resulting from lymphocyte centrifugation were stained with anti-CD4 and anti-CD8 surface antigen markers for 15 min on ice. The cells were washed with PBS and resuspended in flow cytometry buffer. CD8 and CD4 surface marker antibodies were purchased from Biolegend.

### Cell sorting

Prepared splenocytes were stained with biotin-conjugated anti-CD4 antibody on ice for 30 min, washed, and then incubated with magnetic streptavidin for 15 min. After being resuspended in cell-sorting buffer, the CD4^+^ T cells were isolated by negative selection using separate columns. The purity of the resulting CD4^+^ T cells was determined as being >90%. The sorted cells were subsequently used for intracellular staining of p62/sequestosome (SQSTM)1, light chain (LC)3B, and apoptosis markers, western blotting, quantitative real-time polymerase chain reaction (qPCR), and transmission electron microscopy (TEM).

### Autophagy marker：intracellular p62/SQSTM1 and LC3B staining

Intracellular p62/SQSTM1 and LC3B staining was performed using a Foxp3/Transcription Factor Staining Buffer Set (eBioscience) to permeabilize the cells. After being washed, the cells were incubated with anti-p62/SQSTM1 (Abcam) and anti-LC3B (Abcam) antibodies on ice for 15 min, followed by incubation with secondary antibodies on ice for 15 min.

### TEM for autophagy flux

CD4^+^ T cells separated using the cell-sorting procedure described above were fixed with 4% paraformaldehyde, 2.5% glutaraldehyde in sodium phosphate buffer (0.1 M, pH 7.2) overnight at 4°C, and post-fixed with 1% OsO_4_ in distilled water for 1 h at room temperature. Prior to dehydration, the lymphocytes were embedded in 2% agar for collection. After dehydration via graded ethanol, the samples were embedded in Epon 812. Ultrathin sections were cut with an ultramicrotome (Ultracut E), stained with aqueous uranyl acetate and lead citrate, and observed under a transmission electron microscope (JEM1230; Jeol). Autophagosomes were defined as double-membraned structures that enclosed cytoplasm with damaged organelles in various stages of degradation. Autolysosomes were defined as single-membraned vesicles with cytoplasmic or organellar debris in various stages of degradation.

### Apoptosis assay

The cells were stained with an Annexin V/FITC Detection Kit (BD) and propidium iodide (PI) (BD) and examined by FACS Calibur (BD) using Cell Quest Pro software at an excitation wavelength of 488 nm and emission wavelength of 530 nm. A minimum of 10,000 cells was analyzed per sample and illustrated as a dot plot using Flowjo software.

### Western blotting

Proteins were extracted from cells using RIPA buffer. After centrifugation for 15 min at 4°C at 14,000 × *g*, the resulting upper supernatant was collected, and its protein was measured using the bicinchoninic acid method. Thirty micrograms of protein was subjected to SDS-PAGE and transferred to polyvinylidene difluoride membranes. After blocking with 5% nonfat milk, the membranes were incubated with specific primary antibodies overnight. The membranes were washed three times with TBST buffer, incubated with the corresponding horseradish-peroxidase-conjugated secondary antibody for 1 h, and then developed on X-ray films using chemiluminescent reagents. Images were captured by Bio-Rad ChemiDoc XRS+, and densitometric analyses were performed using Quantity-One software (Bio-Rad). The antibodies used in this study were: anti-phospho (p)-mTOR (Cell Signaling Technology) and anti-p-p70S6 (Cell Signaling Technology); anti-glyceraldehyde-3-phosphate dehydrogenase (GAPDH) (Santa Cruz Biotechnology) was used as a control.

### Quantitative real-time polymerase chain reaction

Total RNA was extracted from sorted CD4^+^ T cells using TRIzol Reagent (Invitrogen). The IQ5 detection system and SYBR Green Real time PCR Master Mix were used for qPCR analysis. The conditions of qPCR were as follows: pre-denaturation for 5 min at 95°C, denaturation for 30 s at 90°C, annealing for 40 s at 60°C, and extension for 40 s at 72°C, for a total of 40 cycles. The PCR primers used were: *BIM*: 5ʹ-GAGATACGGATTGCACAGGA-3ʹ and 5ʹ-TCAGCCTCGCGGTAATCATT-3ʹ. *ACTIN* was used as an internal control, and fold changes were calculated by relative quantification (2^−△△Ct^). Each experiment was conducted three times.

### Statistical analysis

Data were analyzed using SPSS version 18.0 software. All data for continuous variables in this study had normal distributions and are shown as the mean ± standard deviation (SD). Differences were assessed using analysis of variance followed by the least significant difference test. Survival analysis was performed by Kaplan–Meier analysis followed by the log-rank test. A *p* value <0.05 was considered to be statistically significant.

## Results

### Construction of mouse model of lethal sepsis by C. albicans bloodstream infection

**Figure 1.**
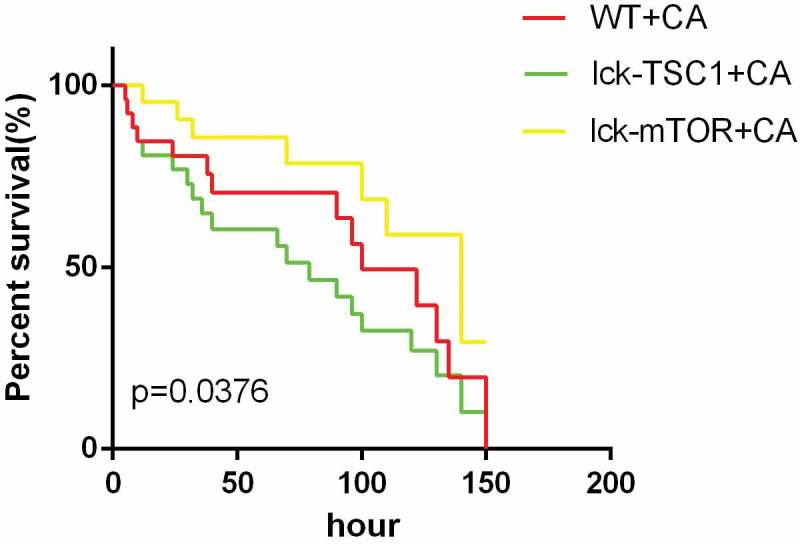
Survival rates of fungal sepsis mice. Survival rates between WT+CA, lck-mTOR+CA, and lck-TSC1+ CA mice. Survival rates were observed every 2 h in the first 12 h after *Candida* injection and then observed every 6 h. n = 20–25 mice per group. p < 0.05 was significant, analyzed by log-rank test. Median survival time of WT+CA was 100h. CA =* Candida albicans*; WT = wild type.

**Figure 2.**
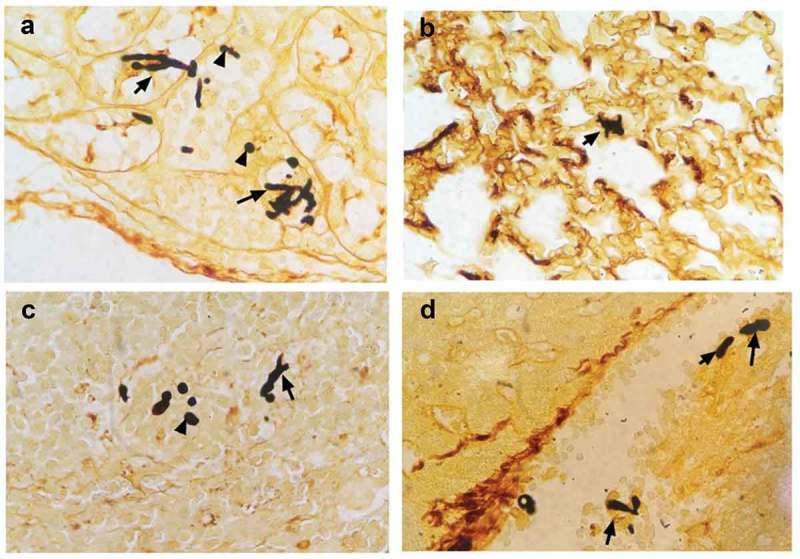
Severe invasive *C. albicans* multiple organs infection mouse model was successfully constructed. Representative kidney (a), lung (b), spleen (c) and liver (d) histological sections from 12 h post-infection were stained with GMS to visualize *C. albicans* hyphae and spores. Black arrows indicate hyphae; black arrowhead indicates a spore. Magnification, 400 × .

We constructed a mouse model of rapidly progressive lethal sepsis induced by *C. albicans* bloodstream infection to mimic the clinical characteristics of *C. albicans* septic shock. To this end, we upgraded the candida intravenous challenge model [25,26], which is one of the recognized models that can mimic clinical candidemia. The severity of this model is directly related to the variety and sex of the mice used, as well as the variety and dose of the strains of *Candida* used in the challenge [27,28]. It has been demonstrated that the median survival time of mice has an approximately linear relationship with the severity of the disease model, which is indicated by renal fungal counts at 12 h after challenge [29]. Therefore, we chose a high concentration of 10^6^ cfu to produce lethal *Candida* sepsis and collected relevant data 12 h after challenge. In addition, we use the Murine Sepsis Score (MSS) [30], which is a validated, reliable and independent scoring system for sepsis severity evaluation to further verify our model success [26]. As we expected, after infection, the mice showed typical disease symptoms, like ruffled coat caused by reduced grooming, increased/decreased movement, abnormal posture (hunched back), and trembling. The MSS is more than three during the whole experiment, which means the mice all died from sepsis. The median survival time was 3–5 days (Figure 1), which is consistent with the clinical mortality in septic shock caused by *Candida albicans*. The successful establishment of our mouse model of severe invasive *C. albicans* multiple organs infection was confirmed by GMS staining. We found distribution of *C. albicans* hyphae and spores in the kidneys, spleens, livers and lungs of infected mice (Figure 2). The results indicated that the model simulated well the clinical characteristics of septic shock caused by candidemia.

### mTOR pathway activation was elevated in C. albicans-infected mice compared with uninfected control mice

**Figure 3.**
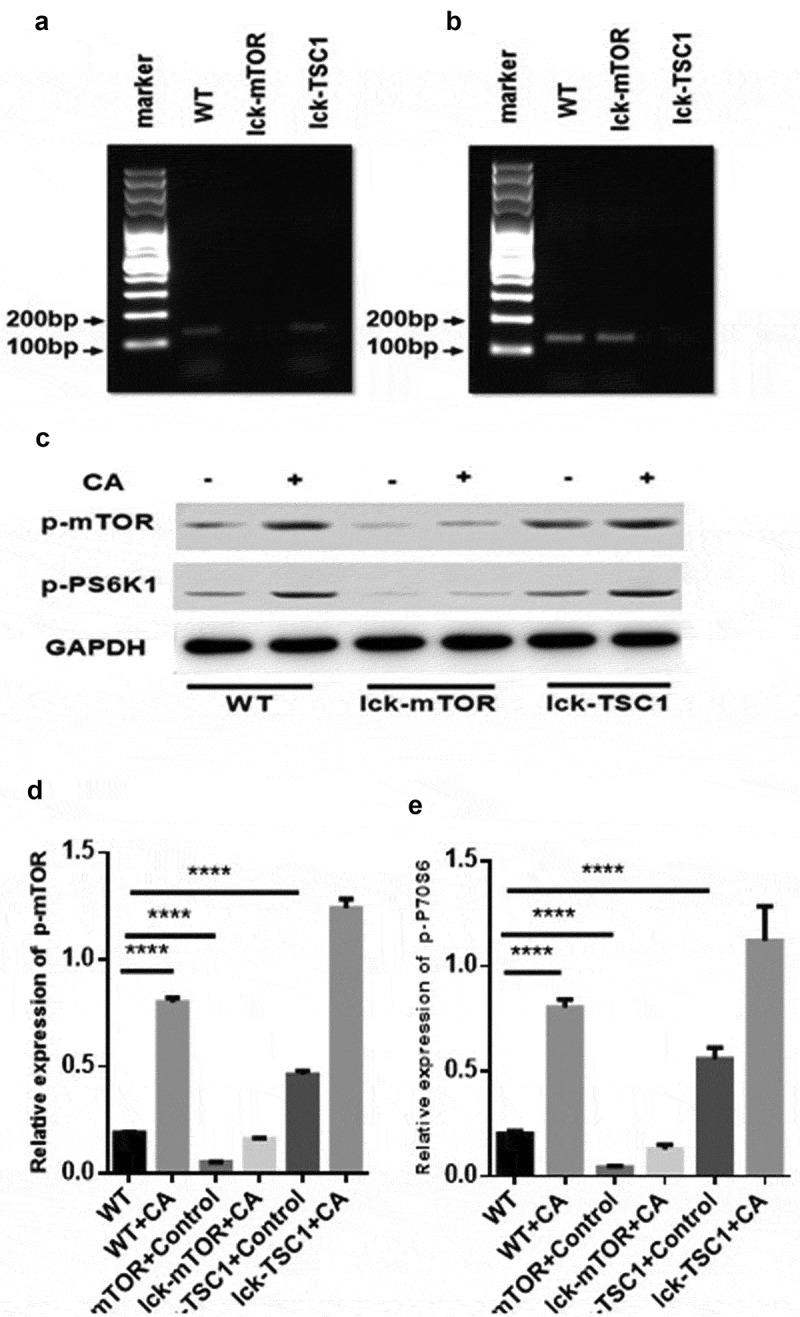
mTOR pathway activation was elevated in fungal sepsis mice compared with uninfected control mice. (a, b) mTOR and TSC1 RNA levels in CD4^+^ T cells. PCR amplification of mTOR (a) and TSC1 (b) RNA from sorted CD4^+^ T cells in WT, lck-mTOR and lck-TSC1 mice, respectively. (c–e) Protein levels of p-mTOR and p-P70S6 in CD4^+^ T cells. Levels of p-mTOR and p-P70S6 in CD4^+^ T cells were quantified by western blotting. The amount of each protein level was normalized by GAPDH. Mean ± SD, six mice per group, ∗p < 0.05, ∗∗∗∗p < 0.0001.

To investigate the activation of the mTOR pathway in CD4^+^ T cells during fungal sepsis, we detected the levels of p-mTOR and p-p70s6 kinase, which were both elevated in *C. albicans*-infected mice compared with uninfected control mice (Figure 3(c,d)). These results indicate that the mTOR pathway might play a vital role in the regulation of CD4^+^ T cells during fungal sepsis.

### Wild-type mice with lethal Candida sepsis had more autophagosomes in CD4^+^ T cells but incomplete autophagy flux compared with uninfected control mice

**Figure 4.**
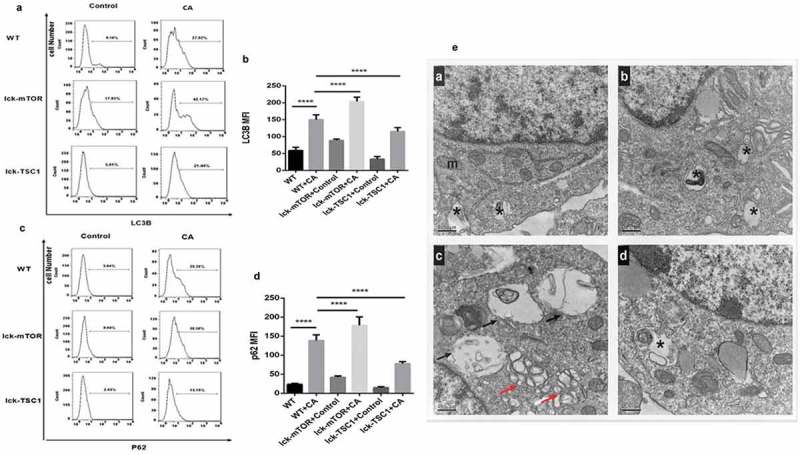
Autophagy process in CD4^+^ T cells in fungal sepsis. (a–d) Autophagy protein expression FACS profiles of LC3B and p62/SQSTM1 staining of CD4^+^ T cells from WT, WT+CA, lck-mTOR+control, lck-mTOR+CA, lck-TSC1+ control or lck-TSC1+ CA mice at 12 h after the procedure. Representative data are shown in A and C. B and D show the MFI of LC3B and p62 staining in CD4^+^ T cells. (e) Ultrastructural features of autophagy vacuoles in CD4^+^ T cells. Autophagy ﬂux was investigated using TEM. (Ea) In control mice, CD4^+^ T cells were normal in appearance, revealing moderate autophagy vacuoles (asterisk) in the cytosol with double- or single-membrane structures containing digested cytoplasmic components. (Eb) WT+CA mice displayed increased autophagic vacuolization. (Ec) Lck-mTOR+CA mice showed more autophagic vacuolization (red arrows) and larger autolysosomes (black arrows). (Ed) Lck-TSC1+ CA mice displayed fewer autophagic vacuoles. Mean ± SD. Six mice per group, ∗∗∗∗*p* < 0.0001. MFI = mean ﬂuorescence intensity.

To evaluate the autophagy level of CD4^+^ T cells in lethal *Candida* sepsis, we assessed the level of the autophagy protein LC3B in CD4^+^ T cells from *Candida*-infected mice. LC3B and p62/SQSTM1 are two major proteins in autophagy. LC3B mainly functions at an early stage of phagophore expansion [31], while p62/SQSTM1 has an adaptor function to recognize ubiquitinated proteins that need to be removed from the cytoplasm during autophagy. The amount of p62/SQSTM1 is generally considered to be inversely correlated with autophagic activity [32,33]. LC3B level in CD4^+^ T cells was significantly higher in mice with lethal *Candida* sepsis compared with that in uninfected control mice (Figure 4(a,b)). The level of P62/SQSTM1, which is a marker protein for incomplete autophagy flux, was also higher in CD4^+^ T cells in *Candida*-infected mice compared with control mice (Figure 4(c,d)). These results suggest that autophagy in CD4^+^ T cells was incomplete in mice with lethal *Candida* sepsis. To confirm these findings, we also investigated the process of autophagy flux using TEM. The number and morphology of autophagosomes and autolysosomes observed in TEM could well reflect the whole autophagy flux [34]. Especially autolysosomes, as the final digestion stage of autophagy are used to represent complete autophagic flux. We found more autophagosomes in *C. albicans*-infected mice than in control mice (Figure 4(e)), while the autolysosomes in *C. albicans*-infected mice were less abundant and larger than those in control mice. This result also implies incomplete autophagy flux in mice with lethal *Candida* sepsis.

### Gene knockout effects of lck-mTOR and lck-TSC1 mice were confirmed by mRNA and protein levels

To further investigate the mechanism mTOR pathway in regulation of CD4^+^ T cells in fungal sepsis, we used two kinds of gene knockout mice: T-cell-specific mTOR knockout (lck-mTOR) mice, and T-cell-specific TSC1 knockout (lck-TSC1) mice. The gene knockout effect was confirmed by mTOR and TSC1 mRNA expression levels (Figure 3(a,b)). There was almost no mTOR mRNA expression in lck-mTOR CD4^+^ T cells and no TSC1 mRNA expression in lck-TSC1 CD4^+^ T cells. This confirmed the effect of gene knockout in lck-mTOR and lck-TSC1 mice. We also confirmed the gene knockout effect at the protein level by detecting p-mTOR (phosphorylation at Ser2448) and activation of p70s6 kinase (phosphorylation at Thr389), which were both elevated in lck-TSC1 mice and both reduced in lck-mTOR mice (Figure 3(c,d)).

### T-cell-specific mTOR deletion ameliorated CD4^+^ T-cell survival in mice with lethal Candida sepsis

**Figure 5.**
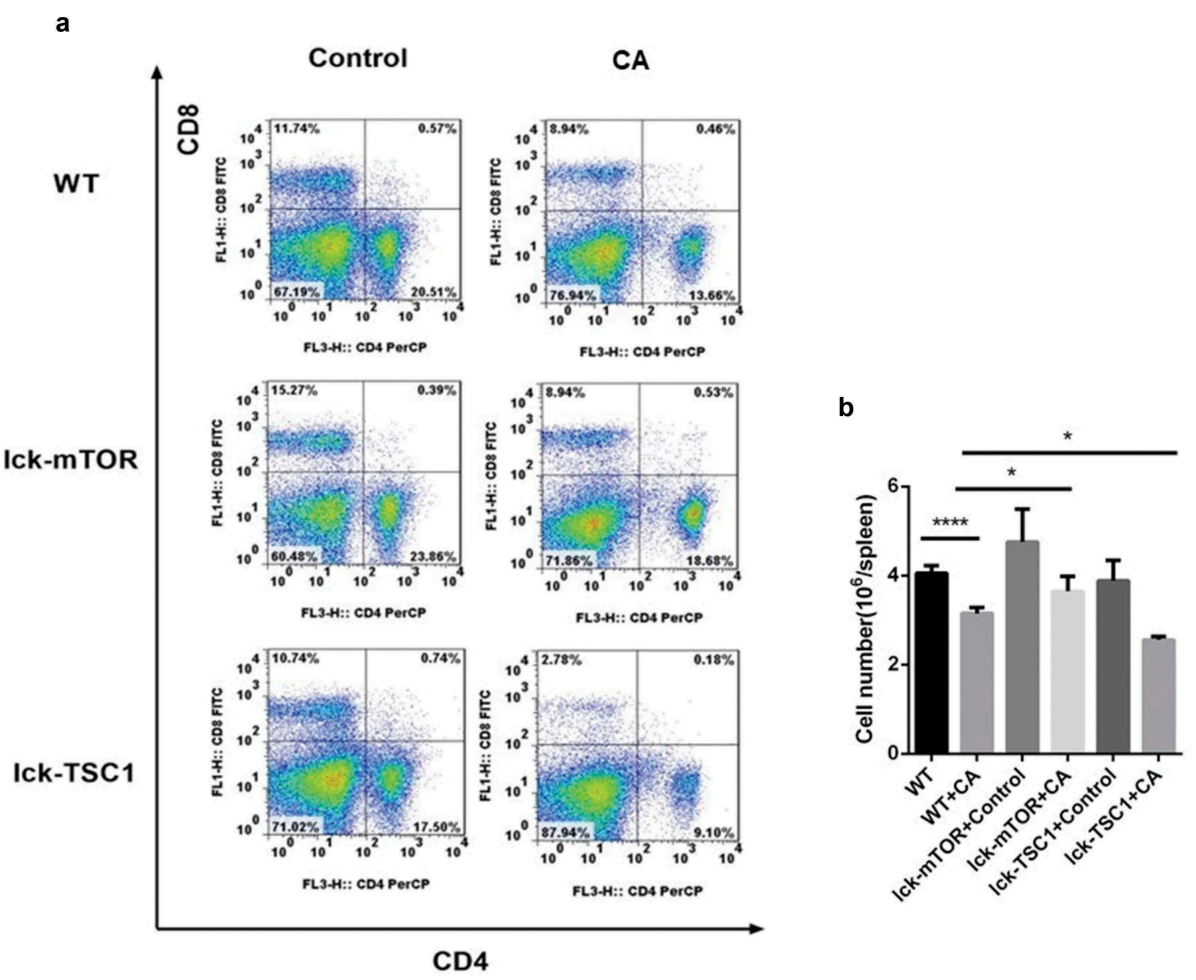
CD4^+^ T cell counts in *Candida*-infected mice. Splenocytes were obtained at 12 h after *Candida* injection. (a) Gating strategy for CD4^+^CD8^−^ and CD4^−^CD8^+^ T cells by flow cytometry analysis. (b) CD4^+^ T cell count for each specimen. Mean ± SD, six mice per group, ∗p < 0.05, ∗∗∗∗p < 0.0001.

To investigate the state of CD4^+^ T-cell survival during lethal *Candida* sepsis, we assessed the percentage of CD4^+^ T cells in the spleens of *Candida*-infected mice. The percentage of CD4^+^ T cells in wild-type mice was significantly lower following *Candida* infection. Compared with *Candida*-infected wild-type mice, mice with T-cell-specific knockout of mTOR that were similarly infected with *Candida* had a greater number of CD4^+^ T cells, whereas *Candida*-infected mice with T-cell-specific knockout of TSC1 had fewer CD4^+^ T cells (Figure 5).

### The mTOR pathway increased CD4^+^ T-cell autophagy in lethal Candida sepsis mainly through increasing the number of autophagosomes

We investigated the role of the mTOR pathway in CD4^+^ T-cell autophagy in lethal *Candida* sepsis. Expression of LC3B following lethal *Candida* sepsis was higher in CD4^+^ T cells from mTOR knockout mice compared with wild-type mice (Figure 4(a,b)). However, inhibition of the mTOR pathway did not decrease expression of P62/SQSTM1 in CD4^+^ T cells (Figure 4(c,d)). We further used TEM to assess autophagy in CD4^+^ T cells and found mTOR knockout mice had a higher number of autophagosomes in their CD4^+^ T cells, while there was no significant difference in the number and size of their autolysosomes (Figure 4(e)). These results indicate that the mTOR pathway enhances CD4^+^ T-cell autophagy in lethal *Candida* sepsis, mainly through increasing the number of autophagosomes rather than by ameliorating incomplete autophagy.

### T-cell-specific mTOR knockout inhibits CD4^+^ T-cell apoptosis in mice with lethal Candida sepsis

**Figure 6.**
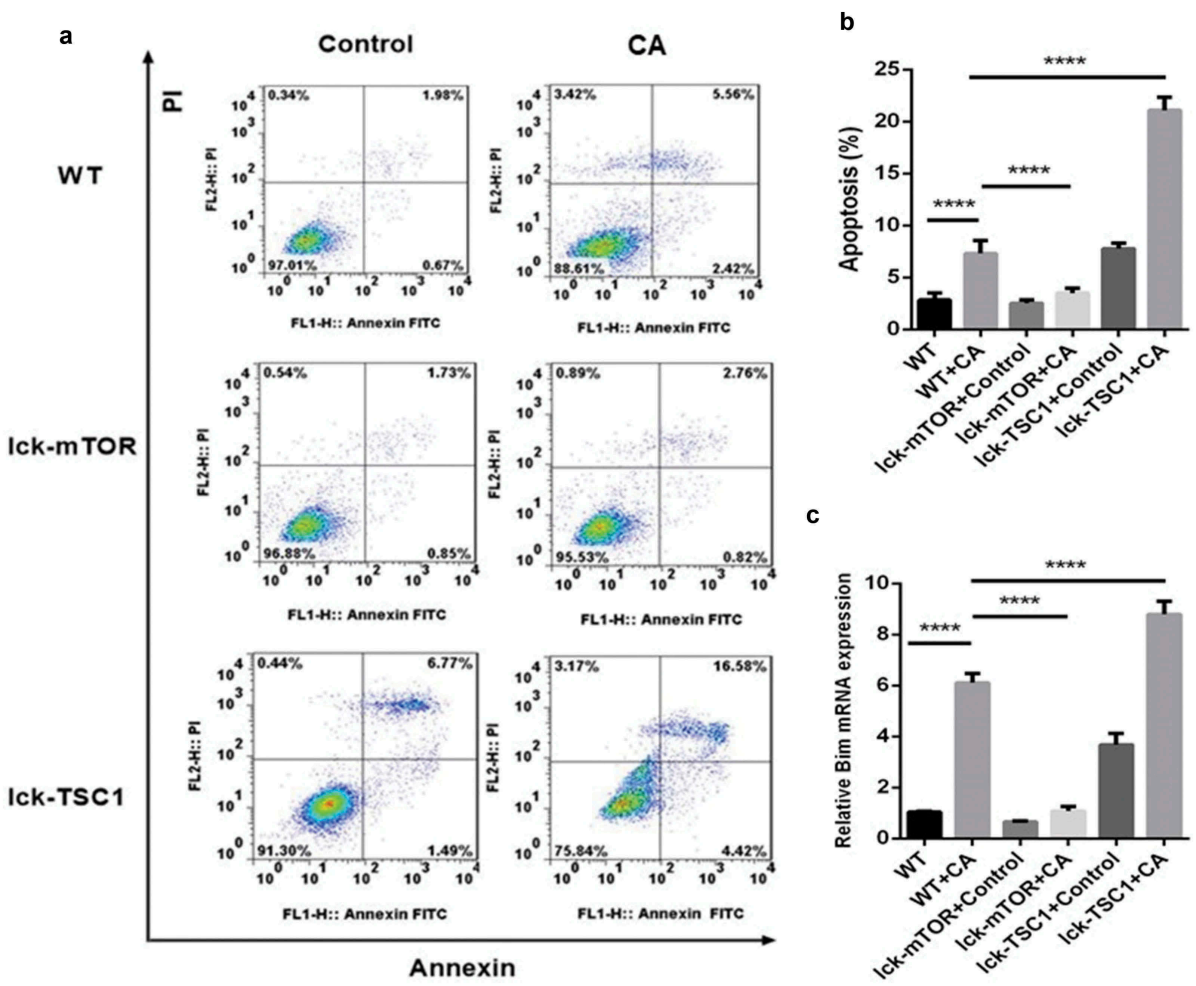
Evaluation of apoptosis in CD4^+^ T cells in fungal sepsis. (a) The subpopulation of annexin-V-positive and PI-negative stained CD4^+^ T cells was considered to be undergoing early apoptosis, while the subpopulation of annexin-V-positive and PI-positive stained CD4^+^ T cells was considered to be undergoing late apoptosis. (b) The apoptosis rate of CD4^+^ T cells, as analyzed by ratio of annexin-V-positive and PI-positive/negative stained CD4^+^ T cells. (c) Relative RNA expression for the apoptotic gene *BIM*. Mean ± SD, six mice per group, ∗∗∗∗p < 0.0001.

CD4^+^ T-cell apoptosis level was higher in mice with lethal *Candida* sepsis, which might explain the decrease in CD4^+^ T cells caused by lethal *Candida* sepsis. Following fungal sepsis, compared with wild-type mice, the number of annexin-V-positive CD4^+^ T cells was lower in the T-cell-specific mTOR knockout mice but greater in the T-cell-specific TSC1 knockout mice (Figure 6(a,b)). These results indicate that inhibition of the mTOR pathway decreases apoptosis of CD4^+^ T cells in mice with lethal *Candida* sepsis. We also confirmed this finding by measuring expression of the apoptotic gene *BIM. BIM* expression was also elevated by lethal *Candida* sepsis in wild-type mice but not in CD4^+^ T-cell-specific mTOR knockout mice (Figure 6(c)).

### T-cell-specific mTOR knockout mice had an increased survival rate in a lethal Candida sepsis model

The lethal *Candida* sepsis mortality rate in the CD4^+^ T-cell-specific mTOR knockout mice was significantly lower compared with control mice, whereas the CD4^+^ T-cell-specific TSC1 knockout mice had significantly higher mortality (Figure 1).

## Discussion

Septic shock caused by fungal infection is characterized by insidious onset, rapid progress, difficult treatment, and high mortality. Our previous study has shown that lymphocyte subsets are closely associated with prognosis during this process, which may partly uncover the difficulty of treatment caused by the immunosuppression that follows systemic infection [35]. The mechanism of immunosuppression in sepsis caused by other pathogens has already been demonstrated, i.e., apoptosis of several immune cell types including macrophages, T cells, B cells, and dendritic cells. CD4^+^ T cells are reported to play a major role in the pathophysiology of immunosuppression [9]. However, the state of immunosuppression in systemic *Candida* infection is not clear. CD4^+^ T cells are known to play a vital role in the adaptive immune response to fungal sepsis, therefore, in the present study, we constructed a model to explore CD4^+^ T-cell survival in lethal fungal sepsis. We measured the number of CD4^+^ T cells in the spleen by flow cytometry at 12 h after *Candida* injection. Both the percentage and specific count of CD4^+^ T cells were significantly lower in wild-type mice with bloodstream infection of *C. albicans* compared with uninfected control mice. These results demonstrate that there is a decrease in CD4^+^ T cells in mice during the early stage of *Candida* infection.

We further investigated the underlying signaling pathway mediating the depletion of CD4^+^ T cells during fungal sepsis. The mTOR signaling pathway is extensively involved in lymphocyte biology, including lymphocyte development, activation and differentiation. A recent study by O’Brien et al. found another critical role for the mTOR pathway in lymphocyte survival. They found that deletion of TSC1 in the murine T cell lineage resulted in a dramatic reduction in the peripheral T cell pool, correlating with increased cell death [18]. Based on these results, we hypothesized that the mTOR pathway might also play a vital role in the survival/depletion of CD4^+^ T cells in fungal sepsis. We found that expression of p-mTOR and p-p70S6 kinase was higher in wild-type mice with bloodstream *C. albicans* infection compared with uninfected control mice, indicating that the mTOR pathway is activated by lethal *Candida* sepsis. *Candida*-infected mice with T-cell-specific mTOR deletion displayed significantly less CD4^+^ T cell depletion, whereas *Candida*-infected mice with T-cell-specific TSC1 deletion mice showed even more CD4^+^ T cell depletion, compared with *Candida*-infected wild-type mice. These results demonstrate that the mTOR pathway is involved in CD4^+^ T-cell survival in fungal sepsis, although the specific mechanism is not revealed.

Autophagy, also known as type 2 programmed cell death [36], is a protein-degradation system that is essential for cellular homeostasis. It is mediated by the mTOR pathway and plays a complex role in cell survival. Moderate autophagy can protect cells from death by providing essential metabolic support during nutrient deprivation, while excessive autophagic degradation can also commit the cells to death [37]. A recent study demonstrated that autophagy is essential for survival of mature T cells, and that inhibition of autophagy in CD4^+^ T cells causes accumulation of mitochondrial reactive oxygen species, leading to increased CD4^+^ T cell apoptosis [22]. This result was verified in another study that found that once autophagy is inhibited during sepsis, apoptosis is promoted [38]. However, the role of autophagy in CD4^+^ T-cell survival during fungal sepsis has not been reported. Based on these previous findings, we speculated that autophagy might be part of the underlying mechanism mediating the function of the mTOR pathway in CD4^+^ T-cell survival.

To investigate this possibility, we first measured expression of autophagy proteins in CD4^+^ T cells from mice with fungal sepsis. According to the guideline [34], LC3B represents the level of autophagy in mammals, and in combination with other indicators such as p62/SQSTM1 and TEM, it fully reflects autophagy flux. We found that expression of both LC3B and p62/SQSTM1 was significantly higher in the bloodstream of mice infected with *C. albicans* compared with uninfected control mice. To explore further the changes in autophagy flux caused by bloodstream infection of *C. albicans*, we examined by TEM the autophagy vesicles in CD4^+^ T cells. Compared with uninfected control mice, *C. albicans*-infected wild-type mice had more autophagosomes, but fewer and larger autolysosomes. These results demonstrate that although the formation of autophagosomes in CD4^+^ T cells is increased by lethal *Candida* sepsis, the process of autophagy flux in these cells remains incomplete.

We next investigated the function of the mTOR pathway in the regulation of autophagy during fungal sepsis. For mice with severe invasive candidiasis, expression of LC3B and p62/SQSTM1 was higher in CD4^+^ T-cell-specific mTOR knockout mice but lower in CD4^+^ T-cell-specific TSC1 knockout mice compared with wild-type mice. We also examined the autophagy process in CD4^+^ T cells during *C. albicans* infection using TEM and found that, compared with wild-type mice, CD4^+^ T-cell-specific mTOR knockout mice had more autophagosomes but fewer and larger autolysosomes. These results suggest that the mTOR pathway mainly regulates the autophagy process in CD4^+^ T cells via mediating formation of autophagosomes rather than by promoting the process of autophagy vesicle degradation. We also found that, compared with wild-type mice, the number of annexin-V-positive CD4^+^ T cells during *C. albicans* infection was lower in CD4^+^ T-cell-specific mTOR knockout mice but was higher in CD4^+^ T-cell-specific TSC1 knockout mice. These results indicate that inhibition of the mTOR pathway can decrease the apoptosis rate of CD4^+^ T cells in mice with lethal *Candida* sepsis. Lastly, we measured expression of the pro-apoptotic gene *BIM*. During lethal *Candida* sepsis, *BIM* expression was lower in CD4^+^ T-cell-specific mTOR knockout mice but was higher in CD4^+^ T-cell-specific TSC1 knockout mice. Therefore, mTOR may regulate survival of CD4^+^ T cells in fungal sepsis partly through the autophagy–apoptosis pathway. Most interestingly, there was a significant difference in mortality between CD4^+^ T-cell-specific mTOR knockout and TSC1 knockout mice and wild-type mice with severe invasive candidiasis. Compared with wild-type mice, T-cell-specific mTOR knockout mice had a higher survival rate after fungal sepsis, whereas T-cell-specific TSC1 knockout mice had a lower survival rate after fungal sepsis. Our study highlighted the role of mTOR in the process of fungal sepsis by regulating autophagy and apoptosis of CD4^+^ T cells, and that CD4^+^ T cells are affected in this process, leading to changes in survival rate. This is consistent with the cecal ligation puncture (CLP) sepsis model [23,24]. Therefore, we did not measure fungal burden in the target organs. In future experiments, we may further explore the relationship between T cell autophagy and fungal infection by detecting fungal burden.

In conclusion, we suggest that the immune response, signaling pathways and targets may be consistent in sepsis caused by bloodstream infection, regardless of whether the pathogen is bacterial, fungal or viral. This is important for intervention in sepsis caused by bloodstream infection and improvement of survival rate. These results suggest a new target and strategy for fungal sepsis immunosuppression.
